# Movement Recognition through Inductive Wireless Links: Investigation of Different Fabrication Techniques

**DOI:** 10.3390/s23187748

**Published:** 2023-09-08

**Authors:** Giuseppina Monti, Luciano Tarricone

**Affiliations:** 1 Department of Engineering for Innovation, University of Salento, 73100 Lecce, Italy; luciano.tarricone@unisalento.it; 2 CNIT—National Inter-University Consortium for Telecommunications, 43124 Parma, Italy

**Keywords:** resonant, inductive coupling, wearable, motion recognition, conductive yarn, conductive non-woven fabric

## Abstract

In this paper, an inductive wireless link for motion recognition is investigated. In order to validate the feasibility of a wearable implementation, the use of three different materials is analyzed: a thin copper wire, a conductive yarn, and a conductive non-woven fabric. Results from the application of the developed devices on an arm are reported and discussed. It is demonstrated that the proposed textile inductive resonant wireless links are well suited for developing a compact wearable system for joint flexion recognition.

## 1. Introduction

Recently, considerable attention has been devoted to wireless links based on inductive resonant (IR) coupling [1,2,3,4].

The basic implementation of IR links consists of two magnetically coupled distributed inductors loaded by two lumped capacitors, realizing the resonance condition at the operating frequency of the link [5,6].

The main application of these links is Wireless Power Transfer (WPT). In fact, compared to links exploiting electromagnetic (far-field WPT) or electrical (capacitive WPT) coupling, IR WPT has the advantage of ensuring robust performance with respect to the electric properties of the propagation channel and the operating environment in general. Accordingly, various IR WPT links have been proposed for both high power (e.g., electric vehicle recharging [7,8]) and low power applications (e.g., medical implant recharging [9,10,11]).

An interesting new application proposed more recently is the use of IR links for joint flexion monitoring [12,13,14]. The basic idea is to exploit the strong dependence of the performance of IR links on the alignment between the transmitter and the receiver; this dependence, which is a problem for WPT applications [15,16,17], can be exploited for movement monitoring.

Human movement monitoring plays a key role in various applications: human–machine interfaces for virtual reality applications [18], monitoring of diseases affecting movements [19], training of athletes [20,21,22], treatment and monitoring of posture problems [23,24], etc.

Consequently, various techniques have already been proposed in the literature for human movement recognition. These techniques can be classified into two main categories: vision-based and sensors-based techniques.

Vision-based techniques exploit optical and/or infrared cameras, they require a line of sight, and are restricted to suitably equipped environments, such as laboratories [25,26,27,28,29,30]. As for sensors-based systems, they are based on a system of sensors (e.g., accelerometers, gyroscopes, and magnetometers) and Inertial Measurement Units (IMUs) [31,32,33,34]. These technologies do not require suitably equipped environments (the sensors are worn by the monitored person) but are subject to measurement errors related to the processing of the data collected by the sensors (e.g., calculating the position by integrating the data provided by an accelerometer).

In consideration of the limitations of the available technologies, a new solution based on IR wireless links deserves to be further investigated. It does not require a line of sight and could overcome problems related to the processing of the measured data, as no integration operations are required. Additionally, it lends itself to the development of a compact wearable system.

Accordingly, this paper aims to investigate the feasibility of developing a wearable IR wireless link for joint flexion monitoring to be integrated into a garment. The focus is on the implementation of the distributed inductors. Different materials and fabrication techniques are analyzed and discussed regarding elbow flexion monitoring. In more detail, the distributed inductors have been fabricated on an elastic fleece shirt by using three different materials: (1) a thin copper wire, (2) a conductive yarn, and (3) a self-adhesive conductive non-woven fabric. The three materials have different electrical properties and are suitable for developing wearable devices with different manufacturing techniques.

In the case of the copper wire and the conductive yarn, the inductors have been hand embroidered on the shirt, while in the case of the conductive fabric, they have been attached. Experimental data referring to the case where the prototypes are applied on a cardboard support to minimize parasitic effects are presented and compared with those obtained by wearing the prototypes. The performed tests demonstrate the feasibility of developing a compact and wearable system for joint flexion monitoring.

The paper is structured as follows. In Section 2, first the working principle of the monitoring system is briefly illustrated, then the analyzed prototypes (materials and fabrication techniques) are presented. In Section 3 the achieved results are presented and discussed. Finally, some conclusions are drawn in Section 4.

## 2. Materials and Methods

### 2.1. IR Wireless Link for Joint Flexion Monitoring: Working Principle

**Figure 1:**
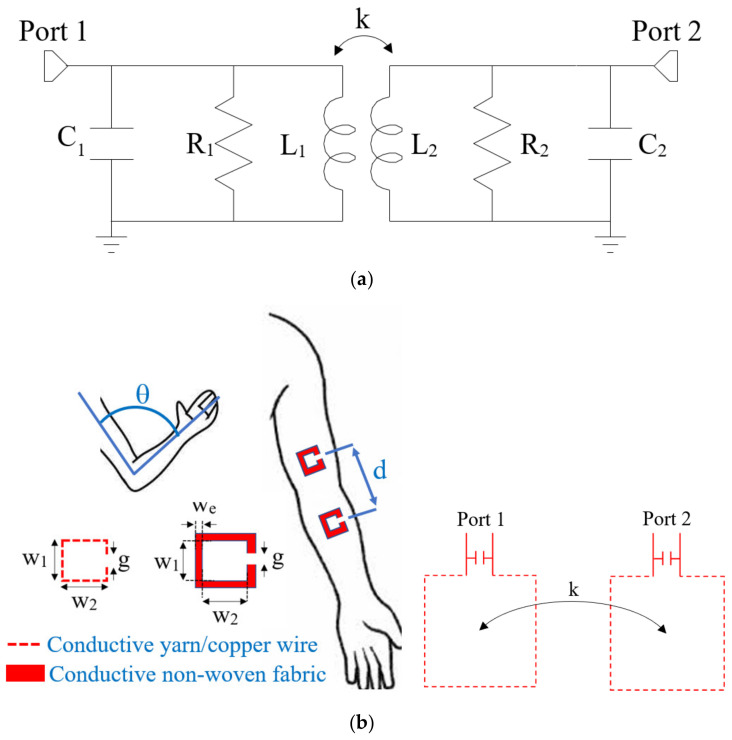
(**a**) Equivalent circuit of an inductive resonant wireless link consisting of two coupled resonators. (**b**) Proposed inductive resonant wireless link for flexion joint monitoring: Positioning of the resonators with respect to the elbow joint and parameters of the distributed inductors.

The equivalent circuit of an inductive resonant wireless link is illustrated in Figure 1a; in its basic configuration it consists of two magnetically coupled resonators: a transmitting and a receiving resonator. Each resonator consists of a distributed inductance loaded by a lumped capacitor in shunt or series configuration. The aim of the lumped capacitors is to obtain the resonance condition at the operating frequency of the link (*f*_0_):

(1) $$f_{0}=\frac{1}{2\pi \sqrt{L_{1}C_{1}}}=\frac{1}{2\pi \sqrt{L_{2}C_{2}}}$$

The resistors *R*_1_ and *R*_2_ model resonator losses which are mainly due to the distributed inductors. The magnetic coupling between the transmitting and the receiving resonator is modeled by the coupling coefficient *k* given by:

(2) $$k=\frac{L_{M}}{\sqrt{L_{1}L_{2}}}$$

where *L_M_* is the mutual inductance. As highlighted in Figure 1a, independently of the specific application, the wireless link can be modeled as a two-port network and analyzed by using one of the possible matrix representations, such as the admittance, the impedance, or the scattering matrix. Assuming a scattering matrix representation, the transmission coefficient of the network (i.e., the scattering parameter *S*_21_) can be used as figure of merit for analyzing the performance in terms of power/data transfer from the input to the output port.

Referring to Figure 1, for given distributed inductances, the coupling coefficient of the inductive resonant wireless link and its transmission coefficient depend on the distance and the alignment between the transmitting and receiving resonators. The basic idea in using an IR wireless link for joint flexion monitoring consists of exploiting this dependence. In fact, by placing the transmitting and receiving resonators before and after the joint so that its flexion affects the alignment of the resonators, it is possible to relate the transmission coefficient of the link to the flexion angle of the joint.

The working mechanism is illustrated in Figure 1b for the case of an elbow. The figure refers to the specific implementation analyzed in this paper. The distributed inductances are implemented as a rectangular planar ring to be applied on the inner surface of the arm and the forearm at a distance *d*.

### 2.2. Analyzed Prototypes: Materials and Fabrication Techniques

As already mentioned, the aim of the present paper is to validate the idea of using an IR wireless link for joint flexion monitoring, focusing on implementations suitable to be embedded into wearable accessories or clothes [35,36]. In this regard, the problems to be overcome are related to the identification of the most suitable materials and the development of a solution guaranteeing the expected performance in the presence of a human body.

**Figure 2:**
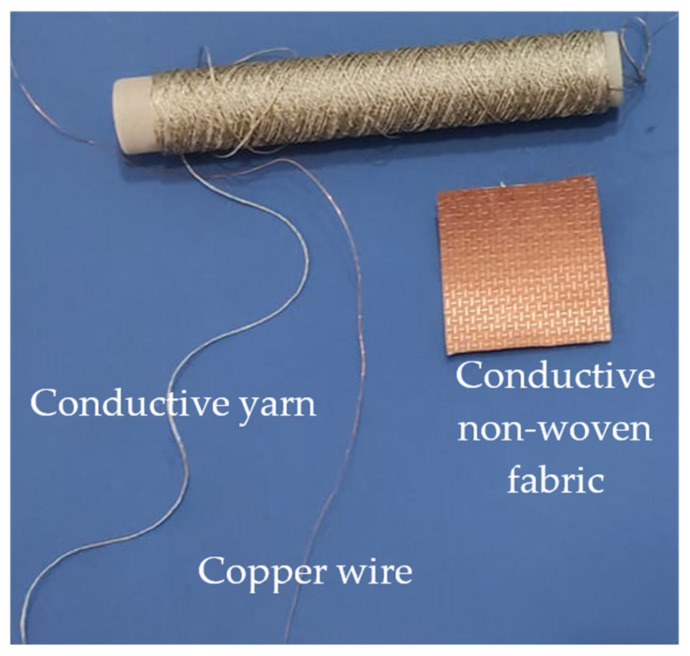
Materials adopted for the fabrication of the distributed inductors: conductive yarn, copper wire, and conductive non-woven fabric.

As far as materials are concerned, they should ensure a transparent integration of the device into a garment; more generally, they should guarantee user comfort and a good fit. In this paper, three different materials have been investigated: a thin copper wire with a radius of 0.2 mm, a conductive yarn, and a non-woven fabric (see Figure 2 and Table 1).

The conductive yarn is the 235/36 HCB by Shieldex [37] with a round cross-section. It is a high conductivity, high-strength, Z-twisted yarn consisting of a metal (99.9% silver)-plated polyamide/nylon yarn with a nitrile rubber coating. Due to the high silver content, the electrical resistivity is lower than 600 Ω/m. The conductive non-woven fabric is the Kiel-SK-96 by Shieldex [38]. It is adhesive, thermally bonded, non-woven, and metallized with pure copper (content of almost 50%). The electrical surface resistance is lower than 0.02 Ohms per square.

Due to the presence of the human body, two different aspects should be taken into account: the presence of biological tissues with non-negligible electrical conductivity and the soft curves of the human body which can lead to deformation of the device (more or less important depending on its dimensions). With regard to the electrical conductivity of biological tissues, the proposed sensor is based on magnetic coupling and has the advantage of a performance independent of the electrical properties of the surrounding environment.

Due to the effect of the curves of the human body, the solution that has been investigated in this paper consists of placing the distributed inductances on the inner surface of the arm; the distributed inductances have been implemented as small rectangular rings so to minimize deformations due to the curves of the arm.

**Figure 3:**
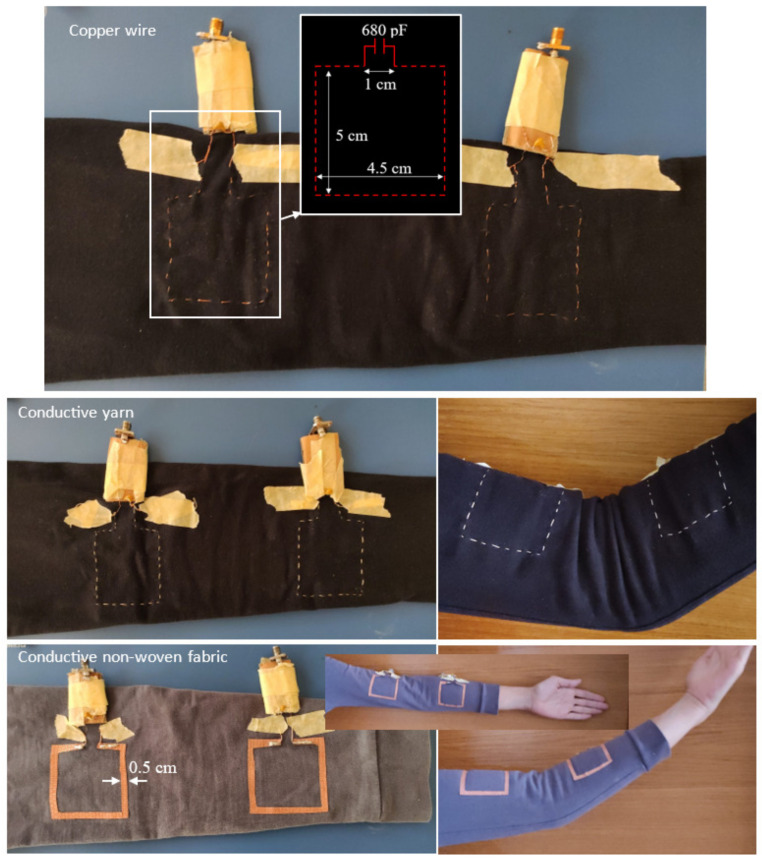
Photographs of the fabricated prototypes. Starting from the top of the figure, prototype fabricated using (1) a copper wire with a radius of 0.2 mm, (2) a conductive yarn, (3) a conductive non-woven fabric. Photographs of the prototypes worn on the arm are also shown.

Photographs of the developed prototypes are shown in Figure 3. Each prototype was fabricated on an elastic fleece shirt. In the cases of the copper wire and the conductive yarn, the coils were hand embroidered, while for the non-woven fabric, the adhesive of the fabric was exploited to attach the coils to the shirt. For all the fabricated prototypes, a copper wire with a radius of 0.2 mm was used for connecting the coils to the lumped capacitors and to SMA (SubMiniature) connectors, which were used for measurements with a Vector Network Analyzer (VNA).

Referring to the parameters illustrated in Figure 1, the dimensions of the fabricated coils are given in Table 2.

## 3. Results

For all the fabricated prototypes, measurements of the scattering parameters were made by using a ZVL VNA by Rode Schwartz. A reference impedance of 50 Ω was assumed at both ports. Two sets of measurements were performed.

The first set of measurements was taken by fixing the prototypes on a planar and rigid cardboard support which was used to simulate elbow flexion. Then, the second set of measurements was taken with the prototypes worn on the arm. The first set of measurements allows to evaluate the performance in an ideal situation; it validates the working mechanism for the developed textile prototypes. The second set of measurements allows to evaluate the effects of the human body on the performance; in fact, the application of the device on the human body (in our tests on the arm and forearm) leads to deformations of the distributed inductors, variations in the distance between the transmitting and the receiving resonators, etc. In both cases, measurements were taken for different values of the flexion angle θ (see Figure 1b).

Measurements on the arm were performed wearing an elastic cotton shirt under the shirt integrated with the monitoring device and were taken on a volunteer subject who was informed and gave written and oral consent to participate in the research.

First, for all the fabricated prototypes, the measured data obtained by using the cardboard support were used to derive the parameters of the equivalent circuit illustrated in Figure 1a. The values of the equivalent circuit parameters were obtained by post-processing the measured data by means of the simulator AWR Design Environment [39].

The calculated values are provided in Table 3. As evident from the values reported in the table, the equivalent inductance obtained for the three prototypes is in the range of 206 nH (non-woven fabric) to 295 nH (copper wire).

For the losses, the highest values were obtained for the inductor fabricated using the conductive yarn, while the lowest values were obtained for the inductor fabricated using the non-woven fabric.

The value of the lumped capacitor was set at 680 pF so as to obtain a frequency of resonance in the range between 10 MHz and 15 MHz. In more detail, for the fabrication of the three prototypes, ceramic capacitors by Huarew were used. According to the data sheet, the capacitance tolerance is 10% (i.e., $C\;\epsilon \;\left[612,\;748\right]$).

**Figure 4:**
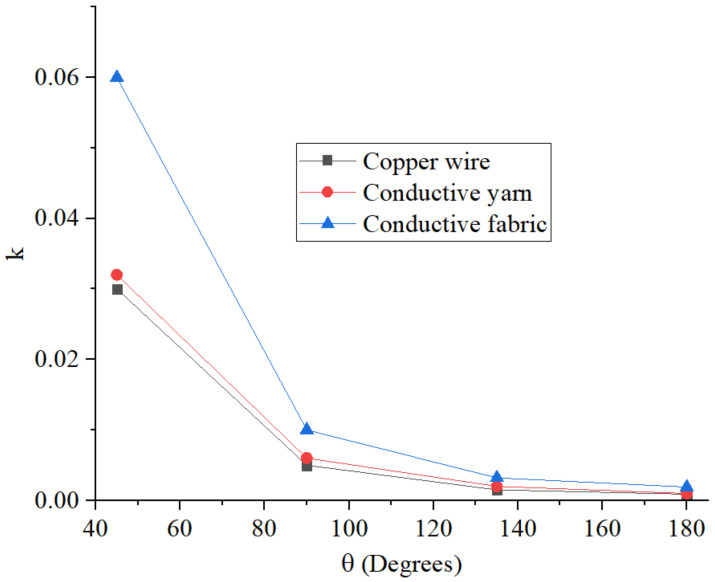
Magnetic coupling coefficient as a function of the flexion angle θ; values calculated for the three prototypes starting from the scattering parameters measured on the cardboard support.

In the table the coupling coefficient calculated for the four values of the flexion angle for which measurements were taken is also reported. For all values of θ, the highest values of the coupling coefficient were obtained for the prototype fabricated using the non-woven fabric, while the lowest values were obtained for the prototype fabricated using the copper wire. The behavior of the coupling coefficient as a function of the flexion angle is illustrated in Figure 4. For all the analyzed cases, it can be seen that the coupling coefficient decreases as θ increases.

**Figure 5:**
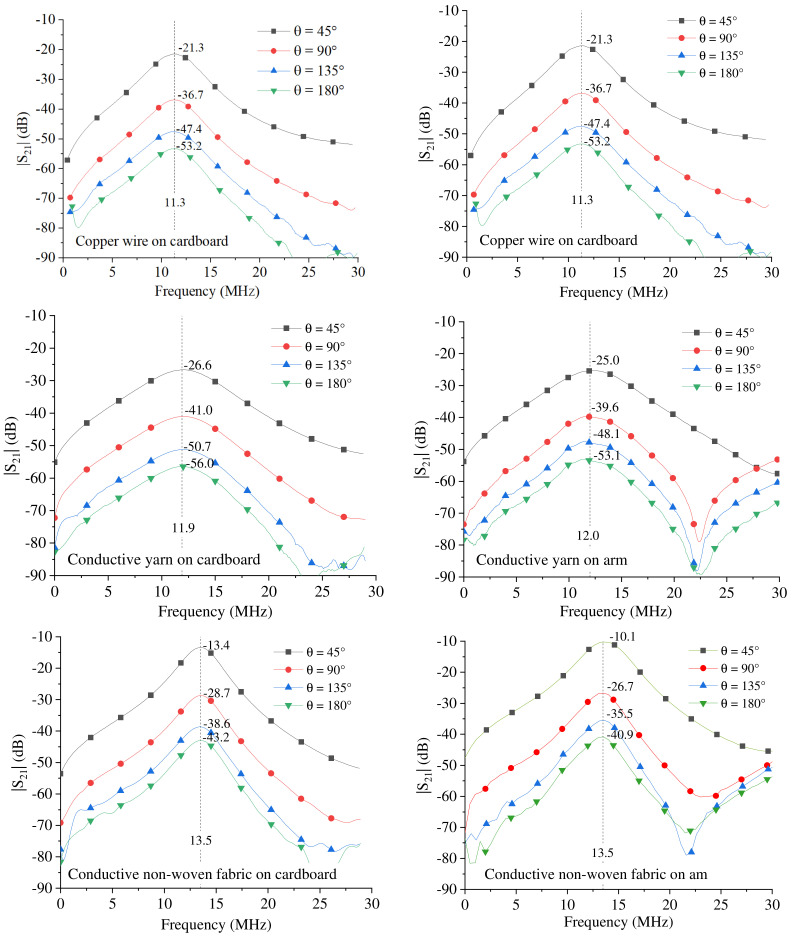
Transmission coefficients measured for the fabricated prototypes. The results obtained using the cardboard support are on the left, while those obtained with the devices worn on the arm are on the right.

The frequency behavior of the measured transmission coefficients is illustrated in Figure 5; in each sub-figure, the dashed line highlights the frequency of resonance. For each curve, the value measured at the frequency of resonance is reported.

By observing the achieved results, it can be seen that for all the prototypes, the amplitude of the transmission coefficient is clearly related to the flexion angle θ.

Additionally, for all the prototypes, it can be seen that the application of the prototypes on the arm does not compromise the operating principle; the same behavior as the measurements on the cardboard is obtained with slightly higher values of the amplitude of the transmission coefficients.

**Figure 6:**
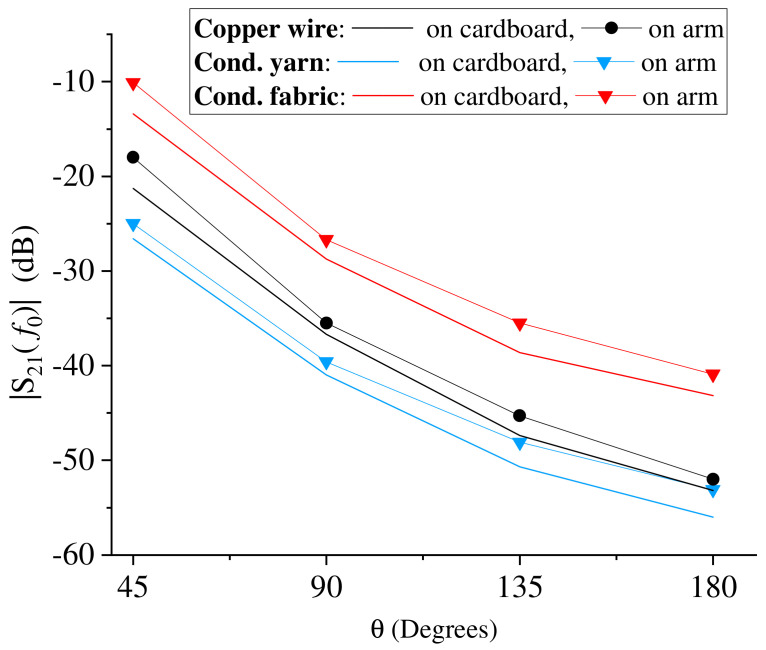
Transmission coefficient as a function of the flexion angle: comparison of the trend obtained for all the prototypes at the frequency of resonance. For each prototype, the trends obtained for both measurements on the cardboard support and on the arm are reported.

This result is highlighted in Figure 6, where the values measured at the frequency of resonance as a function of the flexion angle are reported. The figure compares the results obtained for all the three prototypes on the cardboard support and on the arm. A very similar trend can be observed for all cases.

The figure also highlights the differences obtained for the three prototypes in terms of the amplitude of the transmission coefficient. Due to the lower losses, the conductive fabric provides higher values of the transmission coefficient by about 12 dB with respect to the conductive yarn and by about 8 dB with respect to the copper wire.

**Figure 7:**
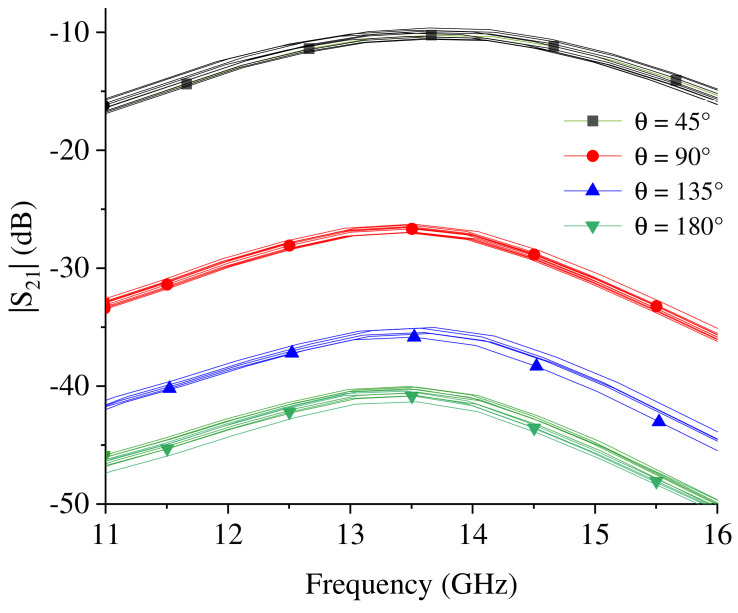
Test performed to verify the repeatability and reproducibility of measurements: results obtained for the prototype fabricated using the conductive fabric. The figure compares the amplitude of the transmission coefficient of ten campaigns of measurements. For each campaign, the prototype was worn and measurements of the transmission coefficient as a function of the flexion angle were taken.

The repeatability and reproducibility of the achieved results was also verified. Tests were performed to verify the effect of small differences in how the sensor is worn (e.g., small differences in the positioning of the resonators with respect to the elbow and to the inner surface of the arm) on the measurements. In particular, 10 measurement campaigns were carried out. In each measurement campaign, the prototypes were worn, and measurements of the scattering parameters were taken by varying the flexion angle. The results obtained for the prototype fabricated using the conductive fabric are illustrated in Figure 7; from the figure, it can be seen that for a given value of θ, small differences can be observed in the amplitudes of the transmission coefficient obtained in the 10 measurement campaigns. However, it is evident that these small differences do not compromise the working principle of the sensor. Similar results were obtained for the prototypes fabricated using the conductive yarn and the copper wire.

Finally, it is worth observing that the results presented in this paper were obtained using the ZVL VNA by Rode Schwartz, not suited to be embedded into a wearable and portable monitoring system; however, this issue can be overcome by using a nano VNA for the measurements of the scattering parameters, such the one adopted in [14,40].

## 4. Conclusions

In this paper, three prototypes for joint flexion monitoring have been proposed and investigated; results referring to the case of elbow flexion monitoring have been presented. The working principle of the analyzed systems consists of exploiting the dependence of the transmission coefficient of an inductive resonant wireless link on the alignment between the transmitting and the receiving resonator.

Three wearable prototypes with inductors fabricated using different materials have been considered: (1) inductors fabricated by using a thin copper wire, (2) inductors fabricated by using a conductive yarn, and (3) inductors fabricated by using a self-adhesive conductive non-woven fabric.

All the prototypes were fabricated on an elastic fleece shirt and experimental data obtained with the prototypes worn on the arm have been presented and discussed.

The achieved results demonstrate that all the analyzed fabrication techniques and materials are well suited for developing a wearable monitoring system for joint flexion monitoring. For all the analyzed prototypes, the transmission coefficient of the inductive resonant wireless link can be related to the flexion angle of the elbow. The best performance in terms of losses, and then in terms of amplitude of the transmission coefficient, was obtained for the prototype fabricated using the conductive non-woven fabric. The prototype with the highest losses is the one fabricated using the conductive yarn; however, this material has the advantage of being suitable for making more complex geometries of the inductors as, in general, it guarantees a greater flexibility in the manufacturing process.

## Figures and Tables

**Table 1:** Materials adopted for the fabrication of the coils (see Figure 2).

Material	Electrical Properties (Resistivity)	Fabrication Technique	Support
Copper wire	0.6 (Ω/m)	Hand embroidery	Elastic fleece shirt
Conductive yarn	<600 Ω/m	Hand embroidery	Elastic fleece shirt
Conductive non-woven fabric	<0.02 Ohms per square	Attached by using the self-adhesive of the fabric	Elastic fleece shirt

**Table 2:** Dimensions of the fabricated coils (refer to Figure 1 for the meaning of the parameters).

w_1_ (cm)	w_2_ (cm)	w_e_ (cm)	g (cm)	d (cm)
5	4.5	0.5	1	15.5

**Table 3:** Values of the parameters of the equivalent circuit illustrated in Figure 1a derived for the three fabricated prototypes.

	L_1_ ≈ L_2_ (nH)	R_1_ ≈ R_2_ (Ω)	C (pF)	k				f_0_ (MHz)
				θ = 45°	θ = 90°	θ = 135°	θ = 180°	
Copper wire	295.00 ± 29.5	160.00	680.00 ± 68	0.0300	0.0050	0.0015	0.0009	11.30
Conductive yarn	266.00 ± 26.6	52.00	680.00 ± 68	0.0320	0.0060	0.002	0.001	11.90
Conductive non-woven fabric	206.00 ± 20.6	180.00	680.00 ± 68	0.0600	0.0100	0.0032	0.0019	13.50

## Data Availability

Not applicable.
